# Association of Iron-Deficiency Anemia and Non-Iron-Deficiency Anemia with Neurobehavioral Development in Children Aged 6–24 Months

**DOI:** 10.3390/nu13103423

**Published:** 2021-09-28

**Authors:** Juan Zheng, Jie Liu, Wenhan Yang

**Affiliations:** 1 Department of Maternal and Child Health, School of Public Health, Sun Yat-Sen University, Guangzhou 510080, China; zhnj3@mail3.sysu.edu.cn; 2 Department of Child Health, Maternity and Child Health Hospital of Baiyun District, Guangzhou 510400, China; liuj6@mail3.sysu.edu.cn; 3 Department of Nutrition and Food Health, School of Public Health, Guangdong Pharmaceutical University, Guangzhou 510006, China

**Keywords:** anemia, children, iron-deficiency anemia, neurobehavioral development

## Abstract

(1) Background: Anemia has comprehensive adverse effects on the growth and development of children. In this study, we analyzed the potential effects of different types of anemia on early-life neurobehavioral development. (2) Methods: A total of 2601 children aged 6–24 months, whose parents agreed to participate in this study, underwent routine blood tests and neurobehavioral development assessment. The children’s parents or other primary caregivers were interviewed with a face-to-face questionnaire at the time of enrollment in the study. Anemia was determined by hemoglobin < 110 g/L and classified into iron-deficiency and non-iron-deficiency anemia according to the levels of serum ferritin, C-reactive protein, and alpha-1-acid glycoprotein. Neurobehavioral development was assessed by the China Developmental Scale for Children and divided into five domains: gross motor, fine movement, adaptability, language, and social behavior. The development quotient (DQ) was used to measure the level of total neurobehavioral development and each domain of neurobehavioral development. (3) Results: The prevalence of anemia in children aged 6–24 months was 26.45%, of which iron-deficiency anemia only accounted for 27.33%. Compared with children without anemia, those with iron-deficiency anemia had a significantly lower developmental quotient (DQ) for total neurobehavioral development and gross motor and adaptability development. The partial regression coefficients were −1.33 (95% CI −2.36, −0.29; *p* = 0.012), −1.88 (95% CI −3.74, −0.03; *p* = 0.047), and 1.48 (95% CI −2.92, −0.05; *p* = 0.042), respectively. Children with non-iron-deficiency anemia had significantly lower DQ for total neurobehavioral development and gross motor and fine movement development than those without anemia. The partial regression coefficients were −0.94 (95% CI −1.64, −0.25; *p* = 0.008), −1.25 (95% CI −2.48, −0.03; *p* = 0.044), and −1.18 (95% CI −2.15, −0.21; *p* = 0.017), respectively. There were no statistically significant differences in total neurobehavioral development and the five domains of neurobehavioral development between children with non-iron-deficiency and iron-deficiency anemia. The partial β values were 0.40 (95% CI −1.53, 2.33; *p* = 0.684), 0.21 (95% CI −1.39, 1.81; *p* = 0.795), 0.63 (95% CI −1.03, 2.28; *p* = 0.457), 0.16 (95% CI −1.78, 2.10; *p* = 0.871), 0.35 (95% CI −1.32, 2.01; *p* = 0.684), and 0.34 (95% CI −0.77, 1.46; *p* = 0.545), respectively. (4) Conclusions: Both iron-deficiency anemia and non-iron-deficiency anemia were negatively correlated with the neurobehavioral development of children. Negative correlations were found between iron-deficiency anemia and gross motor and adaptability development and between non-iron-deficiency anemia and gross motor and fine movement development.

## 1. Background

With the improvement of the economy and living standards, the prevalence of malnutrition has decreased year by year, but anemia is still one of the most serious global public health problems in the 21st century [1]. According to a 2015 WHO report, anemia affected about 800 million children and women in 2011, and the global prevalence of anemia in children aged 6–59 months reached 42.6% [2].

Children aged 6–24 months are at a high risk of anemia. A report on the nutritional development of children aged 0–6 years in China (2012) pointed out that, from 2000 to 2009, the prevalence of anemia in children aged 6–24 months was the highest, and the prevalence in children aged 2–3 years plateaued and gradually decreased after 3 years of age [3].

Anemia has comprehensive adverse effects on the growth and development of children [4,5,6]. Previous studies proved that children with anemia lag behind in physical growth and development to varying degrees [7,8,9]. However, studies related to the effects of anemia on neurobehavioral development were mainly focused on iron-deficiency anemia, and there were no consistent conclusions. For example, one study found that iron-deficiency anemia affected children’s cognitive, motor, socio-emotional, and neurophysiological development [10]. Another study found that iron deficiency affected children’s development in recognition memory, emotion, and motor skills [11]. Some researchers found that iron-deficiency anemia affected the development of children’s fine movement, language, and personal/social skills [12]; other researchers found that iron-deficiency anemia affected the development of children’s adaptive behavior and gross and fine motor skills [13]; and other researchers found that iron-deficiency anemia affected children’s cognition and fine motor and social/emotional skills [14]. The inconsistent conclusions of these studies may be related to differences in the definition of anemia, the methods of evaluating neurobehavioral development, the research objects, or the sample size.

The level of neurobehavioral development in early life is very important for health throughout a person’s lifetime [15]. Therefore, it is crucial to explore the effects of different types of anemia on neurobehavioral development in early life and then implement targeted prevention and early intervention programs. In this research, a cross-sectional study was conducted to analyze the anemia status of children and identify the association of different types of anemia with neurobehavioral development. For this purpose, 2601 children aged 6–24 months, whose parents agreed to participate in the study, underwent physical examinations at the Maternal and Child Health Hospital of Baiyun District, Guangzhou, from 1 January 2018 to 31 December 2019 in order to provide targeted clues and suggestions for the prevention and treatment of anemia.

## 2. Methods

### 2.1. Participants

In this study, 2601 children aged 6–24 months, whose parents agreed to participate in the study, underwent physical examinations in the Child Health Department of the Maternal and Child Health Hospital in Baiyun District, Guangzhou, from 1 January 2018 to 31 December 2019.

The exclusion criteria were as follows: age of less than 6 months or more than 24 months during the study period, premature delivery, low birth weight, congenital malformations or birth defects, perinatal birth injuries, perinatal dystocia or asphyxia, diagnosis of hypoxic-ischemic encephalopathy or any other neurological disease at birth, family genetic history of thalassemia, history of inherited metabolic or chromosome disease, congenital heart disease, and other organic diseases.

The study was conducted according to the guidelines of the Declaration of Helsinki and approved by the Ethics Committee of the School of Public Health, Sun Yat-Sen University.

### 2.2. Blood Specimen Detection Methods

Peripheral blood was collected from the left ring finger at the time the children were enrolled in the study by professional technicians of the laboratory department according to the standard rules of blood specimen collection. Routine blood examination was carried out by a Sysmex XS-1000i automatic five-classification hematology analyzer (Sysmex Corp., Kobe, Japan) to diagnose anemia. A further venous blood test was carried out when the peripheral blood showed anemia to confirm the diagnosis and identify whether it was iron-deficiency anemia or not.

### 2.3. Diagnostic and Classification Criteria of Types of Anemia

Anemia was diagnosed based on the recommendations of the World Health Organization (WHO), United Nations International Children’s Emergency Fund (UNICEF), and United Nations University (UNU): a hemoglobin (Hb) level < 110 g/L in children aged 6–59 months indicates anemia (90–109, 60–89, and <60 g/L correspond to mild, moderate, and severe anemia, respectively) [16,17]. Due to the influence of altitude on Hb, the Hb value for anemia diagnosis needs to be increased by 4% for every 1000 m above sea level. There was no need to adjust Hb in this area because the average altitude in Baiyun District, Guangzhou City, Guangdong Province, is about 10–500 m above sea level.

Classification criteria of the types of anemia: The diagnosis of iron-deficiency anemia (IDA) has three conditions [16,17]: (1) Hb < 110 g/L; (2) blood cell morphology consistent with microcytic hypochromic anemia: mean corpuscular volume (MCV) < 80 fl/L, mean corpuscular hemoglobin (MCH) < 27 pg/L, mean corpuscular hemoglobin concentration (MCHC) < 310 g/L; (3) iron biochemistry test shows iron deficiency, diagnosed according to the health industry standard of the People’s Republic of China (PRC) (WS/T 465-2015) [18]. The boundary value of serum ferritin (SF) to determine iron deficiency needs to be rectified according to C-reactive protein (CRP) and alpha-1-acid glycoprotein (AGP) levels to avoid the false detection of increased serum ferritin in inflammation, infection, or certain diseases: SF < 12 μg/L when CRP ≤ 5 mg/L and AGP ≤ 1 g/L, or SF < 15 μg/L when CRP > 5 mg/L or AGP > 1 g/L, or SF < 22 μg/L when CRP > 5 mg/L and AGP > 1 g/L.

The diagnosis of non-iron-deficiency anemia (NIDA) has two conditions: (1) Hb < 110 g/L and (2) iron biochemistry test shows no iron deficiency: SF ≥ 12 μg/L when CRP ≤ 5 mg/L and AGP ≤ 1 g/L, or SF ≥ 15 μg/L when CRP > 5 mg/L or AGP > 1 g/L, or SF ≥ 22 μg/L when CRP > 5 mg/L and AGP > 1 g/L.

### 2.4. Neurobehavioral Development Assessment

The Developmental Behavior Assessment Scale for Children Aged 0–6 years (WS/T 580-2017) (File S1) [19], which is a health industry standard issued by the National Health and Family Planning Commission of the PRC, and the China Developmental Scale for Children were used to assess the neurobehavioral development of children by standardized trained assessors at the time of enrollment in this study. This scale includes 5 domains of neurobehavioral development: gross motor, fine movement, adaptability, language, and social behavior. The development quotient (DQ) was used to measure the level of total neurobehavioral development and each domain of neurobehavioral development (DQ = mental age/chronological age × 100). Neurobehavioral development was categorized into 5 levels according to the DQ: DQ > 130, 110–129, 80–109, 70–79, and <70, indicating excellent, good, medium, borderline low, and disordered mental development, respectively. A study by Jin reported that the Chinese child development scale has acceptable reliability and validity: the Kendall’s concordance coefficient of the total developmental quotient and of the 5 domains is between 0.98 and 1.000, the Cronbach’s α coefficient (internal concordance coefficient) is between 0.850 and 0.954, the split-half correlation coefficient is between 0.890 and 0.968, the test–retest reliability for children aged 0–4 years old is above 0.900, and it is highly correlated with Gesell and WPPSI-R [20].

### 2.5. Covariates

A questionnaire was administered via face-to-face interviews with the parents or primary caregivers of participants by trained investigators at the time of enrollment in this study, the contents of which were as follows: (1) general information—including infant’s name, sex, and date of birth, parents’ names, contact number, and home address; (2) birth and feeding conditions—including type of delivery (natural childbirth or cesarean section), gestational age at birth, birth weight, birth body length, feeding patterns within 6 months after birth (exclusive breastfeeding, formula feeding, or mixed feeding), time when supplementary food was started, and child’s primary caregivers (parents or others, such as grandparents or nannies); (3) mother’s conditions during pregnancy, such as anemia during pregnancy or not; and (4) other information, such as the annual family income and the education level of the primary caregivers.

### 2.6. Statistical Analysis

EpiData 3.1 and Microsoft Excel were used to process the data. All data were input twice by 2 persons independently, and the consistency and logic were checked by a third person responsible for data curation. Statistical analysis was conducted with SPSS 25.0 (IBM, Chicago, IL, USA). The normality of measurement data was tested by histogram, P-P chart, and single-sample Kolmogorov–Smirnov test (K-S test). The data of normal distribution were expressed as mean ± standard deviation. The data of non-normal distribution were described as median and interquartile interval. The numerical data were described as number of cases (n) and percentage (%). A T-test, one-way ANOVA, and chi-squared test were used to compare the differences between groups. Linear regression was used to analyze the relationship between different types of anemia and neurobehavioral development. The level of statistical significance (*p*-value) was set at 0.05.

## 3. Results

A total of 2601 children aged 6–24 months were investigated in this study. Among them, 1459 (56.09%) were boys and 1142 (43.91%) were girls, 1262 (48.52%) were aged 6–11 months, and 1339 (51.48%) were aged 12–24 months. The overall anemia prevalence was 26.45%. Among children with anemia, mild, moderate, and severe anemia accounted for 94.62, 5.23, and 0.15%, respectively. Iron-deficiency and non-iron-deficiency anemia accounted for 27.33 and 72.67% of all children with anemia, respectively.

### 3.1. Prevalence of Anemia in Children with Different Demographic Characteristics

The prevalence of anemia was 26.18% in boys and 26.80% in girls, 32.73% in children aged 6–11 months, and 20.54% in children aged 12–24 months. Breastfeeding, supplementary food starting at more than 8 months old, primary caregivers other than parents, and primary caregivers having a technical secondary school, senior high school, higher vocational education, or below were associated with a higher prevalence of anemia. There were significant differences in the prevalence of anemia in terms of the children’s age, feeding pattern within 6 months after birth, age when supplementary food was started, type of primary caregivers, and education level of primary caregivers (*χ*^2^ = 49.61, 20.90, 16.13, 29.14, and 9.94, respectively, all *p* < 0.05). There were no significant differences in the prevalence of anemia in terms of sex, gestational age, delivery model, maternal anemia during pregnancy, and annual family income (*χ*^2^ = 0.12, 8.73, 2.03, 0.36, and 0.81, respectively, all *p* > 0.05) (Table 1).

### 3.2. Comparison of Neurobehavioral Development Quotient between Types of Anemia in Children Aged 6–24 Months

According to their anemia status, the children were divided into three groups: no anemia, iron-deficiency anemia, and non-iron-deficiency anemia. The total DQ values for neurobehavioral development of children in the iron-deficiency anemia and non-iron-deficiency anemia groups were significantly lower than those of the group without anemia (*t* = −3.544 and −2.987, all *p* < 0.05). Further pairwise comparison showed that the DQ values of gross motor, fine movement, and adaptability of children in the iron-deficiency anemia group were lower than those in the group without anemia (*t* = −2.683, −2.464, and –2.717, all *p* < 0.05). The DQ values of gross motor and fine movement in the non-iron-deficiency anemia group were lower than those in the group without anemia (*t* = −2.453 and −2.500, all *p* < 0.05). The total DQ for neurobehavioral development and the DQ values of the five domains of neurobehavioral development in the iron-deficiency anemia group were lower than those in the non-iron-deficiency anemia group but with no statistical significance (all *p* > 0.05) (Table 2).

### 3.3. Association of Anemia and Neurobehavioral Development of Children Aged 6–24 Months

To verify the association of anemia with the neurobehavioral development of children aged 6–24 months, we considered the total DQ value for neurobehavioral development, the values of the five domains of neurobehavioral development as dependent variables, and the demographic characteristics as independent variables to build a multiple linear regression model by using the entry method.

An association between anemia and lower neurobehavioral development was observed in children aged 6–24 months. The results of multiple linear regression analysis showed that, after adjusting for age, sex, and other demographic characteristics, the partial regression coefficients (β) for gross motor, fine movement, and adaptability development and total neurobehavioral development of children with anemia, compared with those without anemia, were −1.40 (95% CI −2.47, −0.32), −1.26 (95% CI −2.11, −0.40), −0.90 (95% CI −1.75, −0.05), and −1.05 (95% CI −1.65, −0.44), respectively, and all *p* < 0.05 (Table 3).

Further analysis showed that, after adjusting for age, gender, and other demographic characteristics, the partial β values for gross motor and adaptability development and total neurobehavioral development of children with iron-deficiency anemia, compared with those without anemia, were −1.88 (95% CI −3.74, −0.03; *p* = 0.047), 1.48 (95% CI −2.92, −0.05; *p* = 0.042), and −1.33 (95% CI −2.36, −0.29; *p* = 0.012), respectively. After adjusting for age, sex, and other demographic characteristics, the partial β values for gross motor and fine movement development and total neurobehavioral development of children with non-iron-deficiency anemia, compared with those without anemia, were −1.25 (95% CI −2.48, −0.03; *p* = 0.044), −1.18 (95% CI −2.15, −0.21; *p* = 0.017), and −0.94 (95% CI −1.64, −0.25; *p* = 0.008), respectively (Table 4).

There were no statistically significant differences in total neurobehavioral development and the five domains of neurobehavioral development between children with non-iron deficiency anemia and with iron-deficiency anemia. The partial β values were 0.40 (95% CI −1.53, 2.33; *p* = 0.684), 0.21 (95% CI −1.39, 1.81; *p* = 0.795), 0.63 (95% CI −1.03, 2.28; *p* = 0.457), 0.16 (95% CI −1.78, 2.10; *p* = 0.871), 0.35 (95% CI −1.32, 2.01; *p* = 0.684), and 0.34 (95% CI −0.77, 1.46; *p* = 0.545), respectively.

## 4. Discussion

### 4.1. Prevalence of Anemia in Children Aged 6–24 Months in Baiyun District, Guangzhou

In the present study, the prevalence of anemia in children aged 6–24 months in Baiyun District, Guangzhou, was 26.45%, which was higher than that in Xicheng District, Beijing (6.40%) [21], but lower than that in Huaihua, Hunan Province, China (29.73%) [22], urban India (46.0%) [23], and rural Thailand (41.7%) [24]. However, according to the WHO’s public health grading standard for anemia status in different populations, a prevalence of ≥40% is regarded as a serious public health problem and 20.0–39.9% is a moderate public health problem. The prevalence of anemia in children aged 6–24 months in this area could be considered a moderate public health problem and should receive more attention.

There are many causes of anemia in children, such as nutritional deficiencies (including iron, copper, folic acid, vitamin B12, vitamin A, and so on), acute or chronic inflammation, parasitic infections, immune diseases, malignant tumors, and various chronic diseases. Among them, iron-deficiency anemia is the most common form of anemia. Previous studies found that about 50% of anemia was caused by iron deficiency [5,16,25]. However, the present study found that iron-deficiency anemia accounted for only 27.33% of anemia in this area. The lower proportion of iron-deficiency anemia may be related to the implementation of a government strategy for its prevention and treatment [26,27]. Nutritional supplementation can effectively reduce the risk of anemia in children [28,29,30]. A population-based study in India found that, among 2862 anemic children aged 1–4 years, iron-deficiency anemia (1045, 36.5%) was the most prevalent type, followed by anemia of other causes (not classified as iron-, folate-, or vitamin B12-deficiency anemia) (702, 24.5%), folate- or vitamin B12-deficiency anemia (542, 18.9%), dimorphic anemia (both iron- and folate- or vitamin B12-deficiency anemia) (387, 13.5%), and anemia of inflammation (186, 6.5%) [31]. It is necessary to further analyze the types of anemia and reassess the association of different types of anemia with the neurobehavioral development of children in China.

### 4.2. Association of Anemia with Neurobehavioral Development of Children Aged 6–24 Months

The results show that the DQ value of total neurobehavioral development and the values of gross motor, fine movement, and adaptability development of children with anemia were significantly lower than those of children without anemia, which was consistent with the research of Yang [4], Dai [32], and Kim [33]. The effect of anemia on the neurobehavioral development of children may be related to the decreased oxygen-carrying capacity of hemoglobin to the cerebral blood flow and declined energy metabolism caused by anemia [4]. The hemoglobin in red blood cells is essential for transporting oxygen, and the oxygen supply for developing children may be limited by anemia, which increases the risk of hypoxia. The brain has a huge demand for oxygen. Although the brain accounts for only 2% of body weight, it consumes 20% of the body’s total oxygen [34]. Therefore, the brain is prone to hypoxia or insufficient oxygen supply due to anemia. Hypoxia may interrupt the development of the brain’s nervous system and cause a series of changes and damage to molecules and neurons [35].

The effects of different types of anemia on the neurobehavioral development of children were different in different areas. The results of multiple linear regression analysis show that, after adjusting for age, sex, gestational age, delivery type, maternal anemia during pregnancy, feeding pattern within 6 months after birth, age when supplementary food was started, primary caregivers, educational level of primary caregivers, and annual family income, both iron-deficiency and non-iron deficiency anemia were negatively correlated with gross motor development and total neurobehavioral development of children. Iron-deficiency anemia was also negatively correlated with adaptability, and non-iron deficiency anemia was negatively correlated with fine movement.

Previous research showed that iron deficiency influences oligodendrocytes by affecting the functions of iron-containing enzymes and key enzymes of iron-dependent metabolism, DNA synthesis, respiratory chain, neurotransmitter metabolism, and lipid synthesis, eventually leading to delayed myelin synthesis or hypomyelination, abnormal synaptic structure, and decreased expression of nerve growth factor [36,37]. As a result, the synaptic efficiency, neurotransmitter release, and information conduction velocity of the brain declines, which affects the development of the nervous system. Studies by Doom [38] and Greminger [39] found that iron deficiency could lead to decreased expressions of dopamine D1 and D2 receptors and declined dopamine synthesis in the brain (especially the basal nucleus), which would affect the conduction of dopaminergic neurotransmitters and change dopamine-mediated behavioral activities, consequently leading to varying degrees of decline in cognitive function, emotional changes, and behavior abnormalities. Research by Kennedy [40] and Bastian [37] suggests that iron deficiency affects energy metabolism and cytochrome C oxidase activity in tissues of the whole body, leading to damage to the structural and functional development of the brain, especially the hippocampus, prefrontal cortex, and anterior cingulate cortex. This is related to memory decline (especially long-term spatial working memory), learning ability, and cognitive function. An imbalance in folic acid, copper metabolism, vitamin B12, and vitamin A [41] also influences the development of anemia. Copper mainly exists in the basal ganglia of the brain, hippocampus, cerebellum, various synaptic membranes, and the cell bodies of cortical pyramidal neurons and cerebellar granule neurons. Copper deficiency will seriously affect nerve development [42]. In addition, non-iron-deficiency anemia, such as inflammatory anemia, was also related to lower cognitive function in children [43].

The results also show that there were no statistically significant differences in total neurobehavioral development and the five domains of neurobehavioral development between children with non-iron-deficiency and iron-deficiency anemia. Therefore, we suggest that it is necessary to gradually strengthen the research and prevention of non-iron-deficiency anemia while continuing to strengthen the prevention and treatment of iron-deficiency anemia so as to comprehensively identify and correctly deal with various factors leading to anemia. In addition, it is necessary to strengthen the evaluation of neurobehavioral development in children (especially children with anemia) so as to identify neurobehavioral abnormalities for early intervention and promote the all-around healthy development of children.

## 5. Strengths and Limitations

The main advantage of this study is that it addresses the insufficiency of research on the effect of non-iron-deficiency anemia on the neurobehavioral development of children and confirms that non-iron deficiency anemia is associated with neurobehavioral development. It provides a reference for further in-depth study of the mechanisms of anemia of various causes on the neurobehavioral development of children. We also acknowledge that there are some limitations in our study. First, the questionnaire interviews may have memory bias, and the associations between different types of anemia and neurobehavioral development cannot provide causal inferences by cross-sectional research. Second, non-iron-deficiency anemia could not be further classified according to etiology. Therefore, it is necessary to further develop prospective cohort studies on the effects of anemia of different causes on neurobehavioral development.

## 6. Conclusions

The prevalence of anemia in children aged 6–24 months in Baiyun District, Guangzhou, was 26.45%, of which iron-deficiency anemia accounted for only 27.33%. We found a negative association between both iron-deficiency and non-iron-deficiency anemia and neurobehavioral development in children aged 6–24 months. We also found no statistically significant differences in neurobehavioral development between children with non-iron deficiency and iron-deficiency anemia. Further investigation is warranted to comprehensively identify causality and elucidate the underlying mechanisms of the effect of anemia on neurodevelopment in children.

## Figures and Tables

**Table 1:** Characteristics of 2601 participants aged 6–24 months according to diagnosis of anemia.

Characteristics	Frequency (n)	Number of Anemia Cases (n)	Prevalence of Anemia (95% CI) (%)	*x* ^2^	*p*
Sex				0.12	0.73
Boy	1459	382	26.18 (23.92, 28.44)		
Girl	1142	306	26.80 (24.22, 29.37)		
Age groups				49.61	<0.001 *
6–11 months	1262	413	32.73 (30.13, 35.32)		
12–24 months	1339	275	20.54 (18.37, 22.70)		
Gestational age				8.73	0.12
37 weeks	207	71	34.30 (27.78, 40.82)		
38 weeks	579	152	26.25 (22.66, 29.85)		
39 weeks	973	251	25.80 (23.04, 28.55)		
40 weeks	656	173	26.37 (22.99, 29.75)		
41 weeks	180	40	22.22 (16.09, 28.35)		
42 weeks	6	1	16.67 (15.94, 17.40)		
Type of delivery				2.03	0.16
Natural childbirth	1866	508	27.22 (25.20, 29.25)		
Cesarean section	735	180	24.49 (21.37, 27.61)		
Maternal anemia during pregnancy				0.36	0.55
No	2010	526	26.17 (24.25, 28.09)		
Yes	591	162	27.41 (23.80, 31.02)		
Feeding pattern within 6 months after birth				20.9	<0.001 *
Breastfeeding	676	203	30.03 (26.57, 33.49)		
Mixed feeding	853	252	29.54 (26.47, 32.61)		
Artificial feeding	1072	233	21.74 (19.26, 24.21)		
Age when supplementary food was started				16.13	<0.001 *
<4 months	155	32	20.65 (14.20, 27.09)		
4–5 months	784	181	23.09 (20.13, 26.04)		
6–8 months	1601	450	28.11 (25.90, 30.01)		
>8 months	61	25	40.98 (28.28, 53.68)		
Type of primary caregivers				29.14	<0.001 *
Parents	1614	368	22.80 (20.75, 24.85)		
Others	987	320	32.42 (29.50, 35.35)		
Education level of primary caregivers				9.94	0.007 *
Junior high school and below	1364	376	27.57 (25.19, 29.94)		
Technical secondary school, senior, high school, and higher vocational	642	184	28.66 (25.15, 32.17)		
College, bachelor’s degree or above	595	128	21.51 (18.20, 24.82)		
Annual family income (RMB/year)				0.81	0.67
<100,000	629	171	27.19 (23.70, 30.67)		
100,000–200,000	1312	351	26.75 (24.35, 29.15)		
>200,000	660	166	25.15 (21.83, 28.47)		

* *p* < 0.05. CI, confidence interval.

**Table 2:** Comparison of quotient scores of overall and specific domains of neurobehavioral development in children with different anemia types.

Groups	No. of Cases	Gross Motor	Fine Movement	Adaptability	Language	Social Behavior	Total Development Quotient
Group 1 (no anemia)	1913	95.34 ± 12.35	92.78 ± 9.69	93.98 ± 9.53	91.62 ± 11.06	91.60 ± 10.14	93.07 ± 6.91
Group 2 (iron-deficiency anemia)	188	93.19 ± 10.30	91.21 ± 8.19	92.28 ± 8.03	90.55 ± 9.89	90.54 ± 8.38	91.56 ± 5.42
Group 3 (non-iron-deficiency anemia)	500	93.89 ± 11.71	91.56 ± 9.79	93.17 ± 10.26	90.57 ± 11.97	90.96 ± 10.36	92.02 ± 6.97
F-value		4.93	4.83	3.65	2.25	1.52	7.77
*p*-value		0.007 *	0.008 *	0.03 *	0.11	0.22	<0.01 *
*p*-value (group 2 vs. group 1)		0.02 *	0.04 *	0.02 *	0.42	0.29	<0.01 *
*p*-value (group 3 vs. group 1)		0.04 *	0.04 *	0.09	0.22	0.53	0.009 *
*p*-value (group 3 vs. group 2)		0.83	0.95	0.28	0.90	0.77	0.73

* *p* < 0.05.

**Table 3:** Multiple linear regression analysis of effect of anemia on neurobehavioral development of children aged 6–24 months.

Subjects	β (95% CI)		*p*-Value
	No Anemia	Anemia	
Gross motor			
Cases/Total	1913/2601	688/2601	
Model 1	△	−1.56 (−2.62, −0.49)	0.004 *
Model 2	△	−1.40 (−2.47, −0.32)	0.01 *
Fine movement			
Cases/Total	1913/2601	688/2601	
Model 1	△	−1.24 (−2.09, −0.40)	0.004 *
Model 2	△	−1.26 (−2.11, −0.40)	0.004 *
Adaptability			
Cases/Total	1913/2601	688/2601	
Model 1	△	−0.89 (−1.73, −0.05)	0.04 *
Model 2	△	−0.90 (−1.75, −0.05)	0.04 *
Language			
Cases/Total	1913/2601	688/2601	
Model 1	△	−1.01 (−2.00, −0.03)	0.04 *
Model 2	△	−0.96 (−1.95, 0.04)	0.06
Social behavior			
Cases/Total	1913/2601	688/2601	
Model 1	△	−0.75 (−1.63, 0.14)	0.10
Model 2	△	−0.67 (−1.56, 0.23)	0.15
Total development quotient			
Cases/Total	1913/2601	688/2601	
Model 1	△	−1.10 (−1.70, −0.50)	<0.001 *
Model 2	△	−1.05 (−1.65, −0.44)	0.001 *

Model 1 adjusted for age and sex. Model 2 adjusted for age, sex, gestational age, delivery type, maternal anemia during pregnancy, feeding pattern within 6 months after birth, age when supplementary food was started, primary caregivers, educational level of primary caregivers, and annual family income. Δ indicates control group. * *p* < 0.05. CI, confidence interval.

**Table 4:** Multiple linear regression analysis of effects of anemia types on neurobehavioral development of children aged 6–24 months.

Subjects	No Anemia	Iron-Deficiency Anemia		Non-Iron-Deficiency Anemia	
		Β (95% CI)	*p* Value	Β (95% CI)	*p* Value
Cases/Total	1913/2601	188/2601		500/2601	
Gross motor					
Model 1	△	−2.02 (−3.86, −0.17)	0.03 *	−1.41 (−2.62, −0.20)	0.02 *
Model 2	△	−1.88 (−3.74, −0.03)	0.04 *	−1.25 (−2.48, −0.03)	0.04 *
Fine movement					
Model 1	△	−1.41 (−2.86, 0.04)	0.06	−1.17 (−2.13, −0.21)	0.02 *
Model 2	△	−1.45 (−2.91, 0.003)	0.05	−1.18 (−2.15, −0.21)	0.02 *
Adaptability					
Model 1	△	−1.46 (−2.88, −0.03)	0.04 *	−0.69 (−1.65, 0.27)	0.16
Model 2	△	−1.48 (−2.92, −0.05)	0.04 *	−0.70 (−1.66, 0.27)	0.16
Language					
Model 1	△	−0.90 (−2.56, 0.76)	0.29	−1.02 (−2.13, 0.10)	0.07
Model 2	△	−0.87 (−2.54, 0.80)	0.31	−0.96 (−2.08, 0.17)	0.1
Social behavior					
Model 1	△	−1.02 (−2.53, 0.50)	0.19	−0.64 (−1.65, 0.37)	0.21
Model 2	△	−0.93 (−2.46, 0.59)	0.23	−0.58 (−1.60, 0.45)	0.27
Total development quotient					
Model 1	△	−1.36 (−2.39, −0.34)	0.009 *	−0.10 (−1.68, −0.31)	0.004 *
Model 2	△	−1.33 (−2.36, −0.29)	0.01 *	−0.94 (−1.64, −0.25)	0.008 *

Model 1 adjusted for age and sex. Model 2 adjusted for age, sex, gestational age, delivery type, maternal anemia during pregnancy, feeding pattern within 6 months after birth, age when supplementary food was started, primary caregivers, educational level of primary caregivers, and annual family income. Δ indicates control group. * *p* < 0.05. CI, confidence interval.

## Data Availability

The data presented in this study are available on request from the corresponding author. The data are not publicly available due to ethical restrictions.

## Supplementary Materials

The following are available online at https://www.mdpi.com/article/10.3390/nu13103423/s1, File S1: The Developmental Behavior Assessment Scale for Children Aged 0–6 Years.
