# Supplementary material for: Association of Iron-Deficiency Anemia and Non-Iron-Deficiency Anemia with Neurobehavioral Development in Children Aged 6–24 Months
Source: Nutrients. 2021 Sep 28;13(10):3423. doi: 10.3390/nu13103423 (PMC8537382; doi:10.3390/nu13103423)
Supplement: Supplementary file 1 [file nutrients-13-03423-s001.zip › nutrients-1366241-supplementary.pdf]

ICS 13.100
C 56

WS

Health Industry Standards of The People's Republic of China

WS/T 580−2017

The Developmental Behavior Assessment Scale for Children Aged 0−6 Years

Developmental scale for children aged 0-6 years

Published on October 12, 2017 Implemented on April 01, 2018

Issued by the National Health and Family Planning Commission of the People's Republic of China

Foreword

This standard was drafted in accordance with the rules given in GB/T 1.1-2009.

This standard was drafted by: Capital Institute of Pediatrics, Beijing Maternal and Child Health Hospital, and Institute of Psychology, Chinese Academy of Sciences.

The main drafters of this standard: Bowen Chen, Chunhua Jin, Ruili Li, Yue Zhang, Lili Zhang, Na Li, Zhenmin Gao, Jiajian Zhang, Jianhong Wang, Xiaoyan Wang, Xiaoguo Zheng, Haitao Gao, Wenhong Song, Weilan Liang, Feng Zhang, Jiannong Shi, Xingming Jin, Yufeng Yang, jin Jing, Aimin Liang, Huishan Wang, Hongwei Ma, Lan Zhang, Huimei Wang, Weimin Zhang, Xueting Gao, Qiyun Du, Ruihua Yang, Heru Wang, Huimin Lu, Qi Xu, Huimin Yang, Lihong Wang, Feng Xiao, Xin Liu, Qianqian Xin, Delu Yin, Tao Yin.

Developmental Behavior Assessment Scale for Children Aged 0-6 Years

1 Range

This standard specifies the evaluation content, test methods, reference range of development quotient and the use of the developmental behavior assessment scale for children aged 0-6 years (under 7 years old).

This standard applies to the assessment of the developmental behavior level of children aged 0-6 years (under 7 years old), and is a diagnostic scale for assessing the level of developmental behavior of children.

2 Terms and definitions

The following terms and definitions apply to this document.

2.1 Attribute

Domains of the scale determination. This scale includes five domains for children's neurobehavioral development: gross motor, fine movement, adaptability, language and social behavior. The gross motor domain refers to body posture, head balance, and the ability to sit, climb, stand, walk, run, and jump; The fine movement domain refers to the ability to use fingers; The language domain refers to the ability to understand language and language expression; The adaptability domain refers to the ability of children to respond to and adapt to their surrounding natural environment and social needs; The social behavior domain refers to the ability to communicate with the surrounding people and the ability to take care of themselves in life.

2.2 Mental age; MA

Psychological age

An indicator reflecting the level of children's intelligence.

Note: In the compiled scale, the test items are grouped by age. If the subject passes the 3-year-old test item, it means that his intelligence age measured by the scale is 3 years old.

2.3 Development quotient; DQ

One of the core indicators used to measure children's mental development level is to measure children's development in the aspects of gross motor, fine movement, adaptability, language and social behavior. The calculation formula is as follows (1).

DQ=Mental age/Actual age*100……………………………… (1)

3 Evaluation content

Including five domains, such as gross motor, fine movement, adaptability, language and social behavior, used to measure the developmental behavior of children and assess their developmental level. There are 8 to 10 test items for each month-age group, for a total of 261 test items. See Appendix A.

4 Testing method

4.1 Testing tools

4.1.1 Evaluation scale

Developmental Behavior Assessment Scale for Children Aged 0-6 Years, See Appendix A.

4.1.2 Auxiliary tools

The examiner uses the standardized test toolbox, the test bed, fence bed, small table, small chair, staircase and other test tools, which were matched with the test scale, for testing.

4.2 Test procedure

4.2.1 Calculate the actual month age

4.2.1.1 First, calculate the age of years, months and days of the participant based on the test date and date of birth of the participant, and then convert the age of years and days to Months. Taking the month age as the unit, which retains one decimal place.

4.2.1.2 Days are converted into months as 30 days = 1.0 months, and years are converted into months as 1 year = 12.0 months.

4.2.2 Mark the main measurement month age

The month age segment closest to the actual month age is the main measurement month age. Marking with △ before the main measurement month age. If the main measurement month age is between the two-month age range on the scale, the smaller month age is selected as the main measurement month age. Premature babies are also marked according to their actual month age, and there is no need to correct the month age.

4.2.3 Test start and end

4.2.3.1 The main measurement month age is the starting month age. The items of the main measurement month age are tested first. Regardless of whether the items of a certain attribute of the main measurement month age pass or not, it needs to be measured forward and backward respectively for another 2 months Age. A total of 5 months of age were tested.

4.2.3.2 If the items of 2 consecutive months in one attribute measured forward from the main measurement month age are all passed, the forward testing of this attribute ends. if any one item of the items of 2 consecutive months measured forward from the main measurement month age is failed to Pass, it is necessary to continue to check forward until the items of 2 consecutive months measured forward from the main measurement month age are all pass.

4.2.3.3 Then, the items of 2 consecutive months are measured backward from the main measurement month age. If the items of 2 consecutive months in one attribute are all failed to pass, the backward testing of this attribute ends. If any one item of the items of 2 consecutive months in one attribute is passed, it is necessary to continue to check backward until the items of 2 consecutive months in one attribute are all failed to pass.

4.2.3.4 All attributes shall be tested in accordance with the requirements of 4.2.3.1 ~ 4.2.3.3.

4.2.4 Recording method

The items which passed in the test shall be indicated by ○; the items which failed to pass in the test shall be indicated by ×.

4.3 Operation method and requirements for passing the test

Please refer to Appendix B for the operation method of the scale and the requirements for passing the test.

4.4 Result calculation

4.4.1 Scoring of each attribute

4.4.1.1 1 month to 12 months old

1.0 point for each attribute. If this attribute contents only one test item, the test item marks as 1.0 point. If this attribute contents two test items, each item marks as 0.5 point.

4.4.1.2 15 months to 36 months old

3.0 points for each attribute. If this attribute contents only one test item, the test item marks as 3.0 points. If this attribute contents two test items, each item marks as 1.5 point.

4.4.1.3 42 months to 84 months old

6.0 points for each attribute, if this attribute contents only one test item, the test item marks as 6.0 points; if this attribute contents two test items, each item marks as 3.0 point.

4.4.2 Calculating mental age

4.4.2.1 Count the scores of the test items that have passed continuously to the highest score (if tested subject has passed the test items for two consecutive months, the test ends, and all the previous items will be passed by default). The scores of the failed items will not be counted. The scores of the passed items (including the items which be passed by default) are added item by item, which is the mental age of the attribute.

4.4.2.2 Add up the scores from the five attributes and divide by 5 to get the total mental age, with one decimal place reserved.

4.4.3 Calculating development quotient

The calculation method of development quotient is shown in formula (1).

5 Development Quotient Reference Range

The reference range of development quotient is as follows: >130 is excellent; 110-129 is good; 80-109 is medium; 70-79 is borderline low; <70 is disorders of mental development.

6 Use of the scale

6.1 Test requirements

6.1.1 The testing environment should be quiet and bright. Children under the age of 4 years are allowed to be accompanied by a parent. Children aged 4 years and above can be accompanied by their parents if they are accompanied by development retardation, poor communication or uncoordinated testing.

6.1.2 The tester shall operate in strict accordance with the operation methods and test passing requirements to avoid the hint, inspiration and induction of the parents of the tested children.

6.1.3 The tester shall be familiar with the operation method and test passing requirements.

6.1.4 The position of the tester shall be correct, the desktop shall be clean, and the tools in the test toolbox shall not be seen by the tested children. Take one from each and put it back after use.

6.1.5 The tester shall receive professional training and obtain relevant qualifications before testing.

6.2 Interpretation of results

6.2.1 The tester who has received professional training should explain and judge the level of development behavior of children based on the comprehensive situation of the child.

6.2.2 The tester should properly explain to the parents the level of children's developmental behavior, especially for children who are development retardation.

Supplementary Materials A

Development Behavior Assessment Scale for Children aged 0−6 years (Child Mental Scale-II)

See Table S.1, Figure S.1, Figure S.2 and Table S.2 for the developmental behavior assessment scale for children aged 0−6 years.

Table S.1 Development Behavior Assessment Scale for Children aged 0−6 years (Child Mental Scale-II)

Project	1 month old	2month old	3month old	4 month old	5 month old
Great sport	□1 head upright for a moment when sitting upright with shoulders supported by the tester	□11 Head upright for a moment When sit up by pulled the wrists	□21 Head stands upright when held straight	□30 Stand for a while when held the armpit	□40 Sit up by pulled the wrist lightly
	□2 Tilt and raise the head when lying prone	□12 head lifting off the bed when lying prone	□22 Head raising 45 degrees when lying prone	□31 Head raising 90 degrees when lying prone	□41 Sit up no assistance with head tilted forward
Fine movement	□3 Clench a fist when touching the palm	□13 Hold the bell stick for a moment	□23 Hold the bell stick for 30s	□32 Watch and shake the flower bell stick	□42 Grab the toys nearby
	□4 Natural state of hands	□14 Thumb separates when tapped lightly *	□24 Put hands together	□33 Trying to grab something	□43 play hands spontaneously
Adaptive capacity	□5 Watch the card with black and white targets*	□15 Watch the big toy immediately	□25 Watch toy on the front of the chest immediately	□34 Eyes contact*	□44 notice the small pellets
	□6 Cross the midline with eyes following the red ball	□16 Eyes turn up and down following the red ball *	□26 Eyes and head turn 180 degrees following the red ball	□35 Yells when happy or dissatisfied [R]	□45 Hold a block and look at another
Language	□7 Make small guttural sound[R]	□17 Pronounce a, o, e or other consonants [R]	□27 Sound when smiling	□36 Babbing in quiet time [R]	□46 Pronunciation when seeing familiar people or toys [R]
	□8 Respond to sound*	□18 Make complex reactions to sounds		□37 Find the sound source	

Social behavior	□9 Staring at the speaker	□19 Smile spontaneously [R]	□28 Laugh when see someone	□38 watch the portrait in the mirror	□47 reaction to the image in the mirror
	□10 Eye tracking people who move around	□20 Respond when being teased	□29 Actively observe the surrounding environment without being teased	□39 happy when see mother or other relatives[R]	□48 See food excitedly [R]
Project	6 month old	7 month old	8 month old	9 month old	10 month old
Great sport	□49 turn over from supine to prone position [R]	□59 Prop up the ground with feet when the armpit is held *	□68 Hold object with both hands to stand up	□77 Walk when hands held	□86 Make protective action *
	□50 Can strike the table	□60 Sit up straight by oneself	□69 Sit alone and rotate freely to reach objects	□78 Crawl on hands and knees	□87 Sit up harmoniously from prone position without assistance and sit stably
Fine motor	□51 Tear and knead paper	□61 Rake to get the pellets	□70 Pinch small pellets with thumb and other fingers	□79 Pinch the pellets with thumb and index finger	□88 Pinch the pellets with the fingertips of the thumb and index finger in coordination, skillfully and quickly
	□52 Touch and grasp the target building block	□62 grasp another building block with the other hand after grasping a building block with one hand	□71 Try to take the third building block after grabbing a building block with each hand	□80 Take out the building blocks from the cup	
Adaptability	□53 Hold a building block in each hand	□63 Pass the first building block to the other hand, and then go to get the second building block	□72 Consciously imitate shaking the bell	□81 Beat two building blocks against each other	□89 Initiatively remove the cup and play the building blocks hidden in the cup
	□54 Looking for fallen toys	□64 Reach for toys in the distance	□73 Try to grasp constantly moved toys with hands	□82 Look for and fiddle with or pinch	□89 Look for something hidden in the box

				the tongue of the bell	
Language	□55 Turn around and look for who calling its name	□65 Unconsciously make "da-da ma-ma" sound [R]	□74 Imitate the sound of coughing and tongue [R]	□83 Make welcoming gestures [R]	□91 Imitate "ma-ma", "ba-ba", "na-na", "zou" and other sounds [R]
	□56 Understand the gesture to hug		□75 Expressing needs with gestures (2/3) [R]	□84 Make a goodbye gesture [R]	
Social behavior	□57 Self-feeding food [R]	□66 Hold feet to play or suck	□76 Understand adult facial expressions	□85 Shake head or push away something no want [R]	□92 Know the names of common objects and people
	□58 Can play hide and seek	□67 Resisted reaction to strangers [R]			□93 Get something as instructed

Project	11 months old	12 months old	15 months old	18 months old	21 months old
Great sport	□94 Stand alone for a while	□103 Stand independently	□112 Walk freely by oneself	□120 Directionless throw a ball	□128 Walk on tiptoe [R]
	□95 Hold an object to squat down and take something	□104 Walk forward with one hand is held			□129 Hold the handrail and walk up the stairs
Fine movement	□96 Consciously put the building blocks into the cup	□105 Hold a pen in whole palm to draw lines	□113 Spontaneously draw	□121 Imitate to draw lines	□130 Thread the crystal thread through the buttonhole
		□106 Try to put the pellets into the vial	□114 Take or pour the pellets out of the bottle		□131 Imitate to pull zipper
Adaptive capacity	□97 Open the square scarf and look for the building blocks wrapped in the square scarf	□107 Cap the bottle	□115 Turn the book twice	□122 Erect four or more building blocks vertically	□132 Erect seven or eight building blocks vertically

	□98 Imitation patting doll		□116 Cover the round box	□123 Put the round building blocks into the template correctly	□133 Know the red
Language	□99 Pronounce a sound consciously [R]	□108 Consciously call Mom and dad [R]	□117 Point out the eyes, ears, mouth, nose and hands	□124 Correctly deliver three building blocks to the asked place	□134 Answer simple questions
	□100 Knows means of "no" [R]	□109 Be willing to share something asked	□118 Speak 3~5 words [R]	□125 Consciously say 10 or more words [R]	□135 Speak sentences of 3~5 words [R]
Social behavior	□101 Drink water with a cup [R]	□110 Actively cooperate to wear clothes [R]	□119 Know to take off socks correctly [R]	□126 Control to relieve shit and urine during the day without pants peed [R]	□136 Freely express personal needs [R]
	□102 Take off hat	□111 Joint attention [R]		□127 Ability to eat with a spoon [R]	□137 Play imaginative Game [R]
Project	24 months old	27 months old	30 months old	33 months old	36 months old
Great sport	□138 Jump off the ground with both feet	□146 Go upstairs by oneself	□156 Stand on one foot for 2S or more	□165 Standing long jump	□174 Jumping with alternating feet
		□147 Go downstairs by oneself			
Fine motor	□139 Thread the crystal thread through the buttonhole and pull the thread out	□148 Imitate to paint vertical lines	□157 Wear 3-5 buttons	□166 Imitate to draw circle	□175 Imitate to draw cross lines
		□149 Insert part or all of the zipper head into the zipper lock hole	□158 Imitate to build a bridge with building blocks	□167 Insert all the zipper head into the zipper lock hole and try to zip up	□176 Ability to screw
Adaptive capacity	□140 Turn the book page by page	□150 Know big and small	□159 Know 1 and many	□168 Erect 10 building blocks vertically	□177 Know the meaning of 3

	□141 Put the round building blocks into the inverted template correctly	□151 Correctly put the building blocks of different shapes into the corresponding formwork holes	□160 Correctly put the building blocks of different shapes into the inverted formwork holes	□169 Execute three commands consecutively	□178 Know two or more colors
Language	□142 Speak two or more sentence of poems or nursery rhymes	□152 Speak sentences of 7~10 words	□161 Tell the names of 10 or more pictures	□170 Tell self-gender correctly	□179 Tell the names of 14 or more pictures
	□143 Ability to speak out the function of bowl, pen, stool and ball	□153 Raise hands or feet according to oral instructions	□162 speak out self-name	□171 Understand the meaning of inside and outside	□180 Speak most pronunciation clearly
Social behavior	□144 Spontaneous or imitative say "hello", "goodbye", etc	□154 Take off clothes or pants without help [R]	□163 Pour water back and forth from two cups without spilling	□172 Ability to wear shoes	□181 Knows "hungry, cold, tired"
	□145 Spontaneously ask "What is this?" [R]	□155 Begin to have the concept of right and wrong	□164 Correctly answer or point out where the peel should be thrown	□173 Ability to unbutton	□182 Ability to buckle

Project	42 months old	48 months old	54 months old	60 months old	66 months old
Great sport	☐183 Go upstairs alternately feet	☐ 193 Stand on one foot for 5s independently	☐203 Stand on one leg for 10 seconds	☐213 Jump on one leg	☐ 222 catch the ball
	□184 Jump down the last step of the stairs with feet together	☐194 Jump down the last step of the stairs with feet together and stand firm	☐204 Walk forward 2 meters by toe to heel	□214 Pedal	□223 Walk back 2 meters by toe to heel

Fine movement	☐185 Build circle and square out of plastic plates	☐195 Imitate to draw square	□205 Fold paper with neat edges	□215 Assemble oval according to the picture	□224 Can write your own name
	☐186 Cut a straight line with scissors	☐196 Assemble screws as shown in picture	☐206 Grab peanuts with chopsticks	□216 Try to cut round	□225 Cut smooth round
Adaptability	☐187 Know the meaning of 5	☐197 Find out 3 different places in two pictures	☐207 Similarity	□217 Find out differences (5)	□226 Stand between trees
	☐188 Know 4 colors	☐198 Tell the missing things of pictures (3/6)	□208 Fill the vacancy of the picture (4/6)	□218 Fill the vacancy of the picture(5/6)	□227 Cross cut apple
Language	☐189 Ability to speak antonyms	☐199 Retell compound sentences	□209 Can wash your mouth	□219 What is your last name?	□228 Know your zodiac
	□190 Tell the shape of the figure (△○ ☐)	□200 Know the use of pot, mobile phone and eyes	□210 Know numbers	□220 Say two kinds of round things	☐229 Count backwords
Social behavior	□191 Ability to wear a coat [R]	□201 Ability to play group games [R]	□211 Know morning and afternoon	□220 Where do you live ?	□230 Why do you take the crosswalk？
	□192 Know to answer why need to wash hands before eating	□202 Ability to distinguish toilets between male and female	□212 Count fingers		□231 Chickens swim in water

Project	72 months old	78 months old	84 months old
Great sport	□232 Jump continuously with elbows	□242 Kick the ball with rope	☐252 Kick the ball with rope continuously

	□233 Bounce the ball(2)	□243 Bounce the ball (5)	□253 Pedal alternately	
Fine movement	□234 Assemble rectangles	□244 Copy hexagon	□254 learn to turn over the rope	
	□235 Copy combined graphics	□245 Try to play slipknot	□255 Play slipknot	
Adaptability	□236 Find out differences(7)	□246 Graphic analogy	□256 Digital analogy	
	□237 Know the left and right	☐ 247 The use of flour	□257 What an animal has no feet?	
Language	□238 Describe the picture contents	□248 Summarize the theme of pictures	□258 Why get a vaccination?	
	□239 Work, window,apple and banana (2/3)	□249 Know the clock	☐259 The common points of sweaters, pants and shoes	
Social behavior	□240 What are the four seasons in a year?	□250 Know the day of the week	☐260 Emergency calls	
	□241 Recognize the logo	□251 Read in the rain	□261 Owls catch mice	

Note 1: the test item marked r indicates that the performance of the item can be obtained by asking parents.
Note 2: the test item marked * indicates that attention needs to be payed if the item fails to pass.
Note 3: specification of the bed for survey: 140cm long, 77cm wide, 143cm high and 63cm high. Note 4: specification of the table for survey: 120cm long, 60cm wide, 75cm high, and the desktop color is dark green.
Note 5: specification of stair for survey: upper platform: a platform composed of two opposite ladders,50cm（length）×60cm（width）×50cm（height） (height from the ground). Base full ladder: the length is 150cm(the length of single ladder base: 75cm). Each step surface:60cm（length）×25cm（width×17cm（height）, 3 steps in total. Unilateral handrail: the length is 90cm and the diameter is 2.5cm. Calculate from the ladder surface, the length of handrail is 40cm and the diameter is 2.5cm.

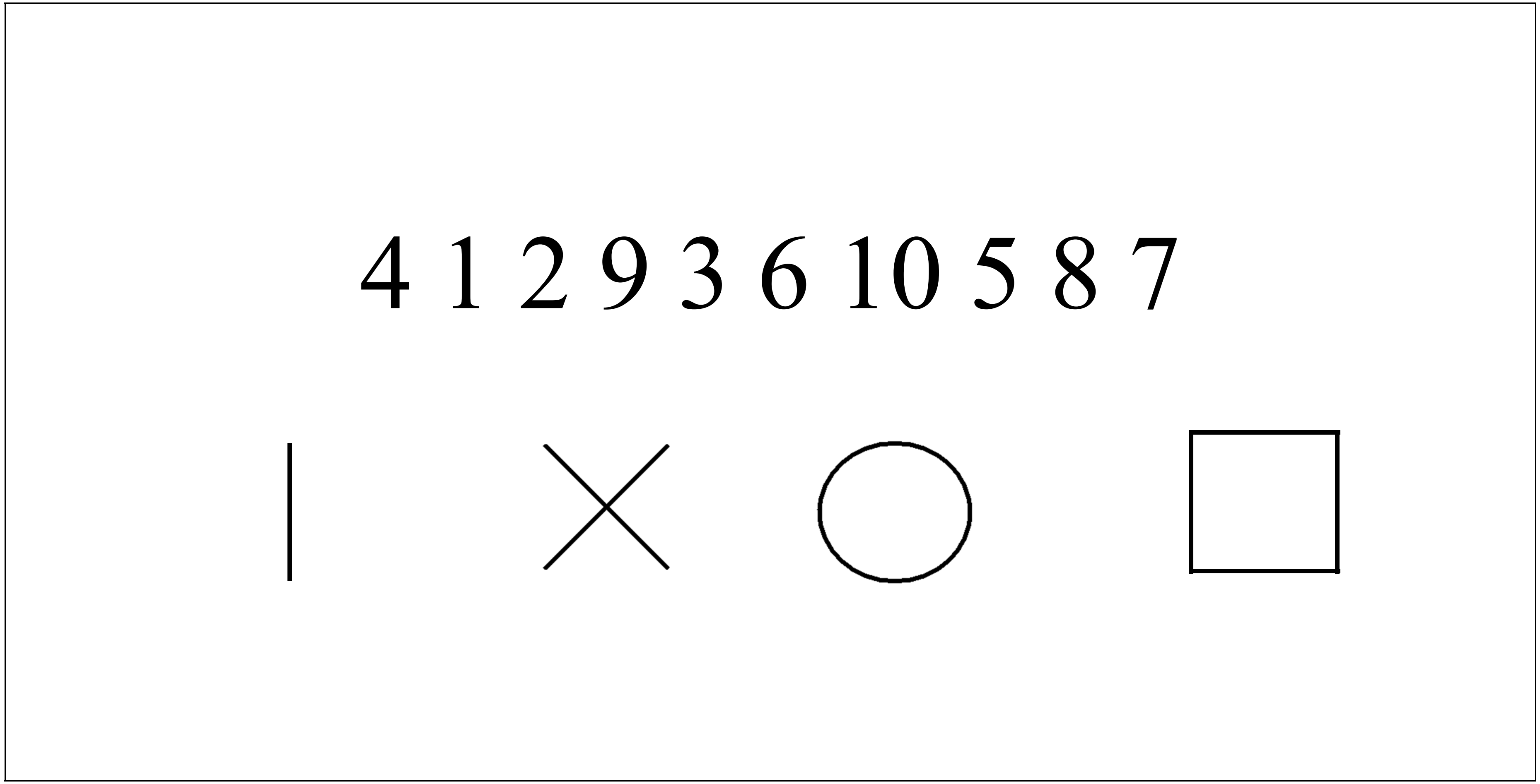

Figure S.1 The Developmental Behavior Assessment Scale for Children from 0−6 Years (Child Mental Scale-II) Digital Recognition and Imitation Drawing Survey

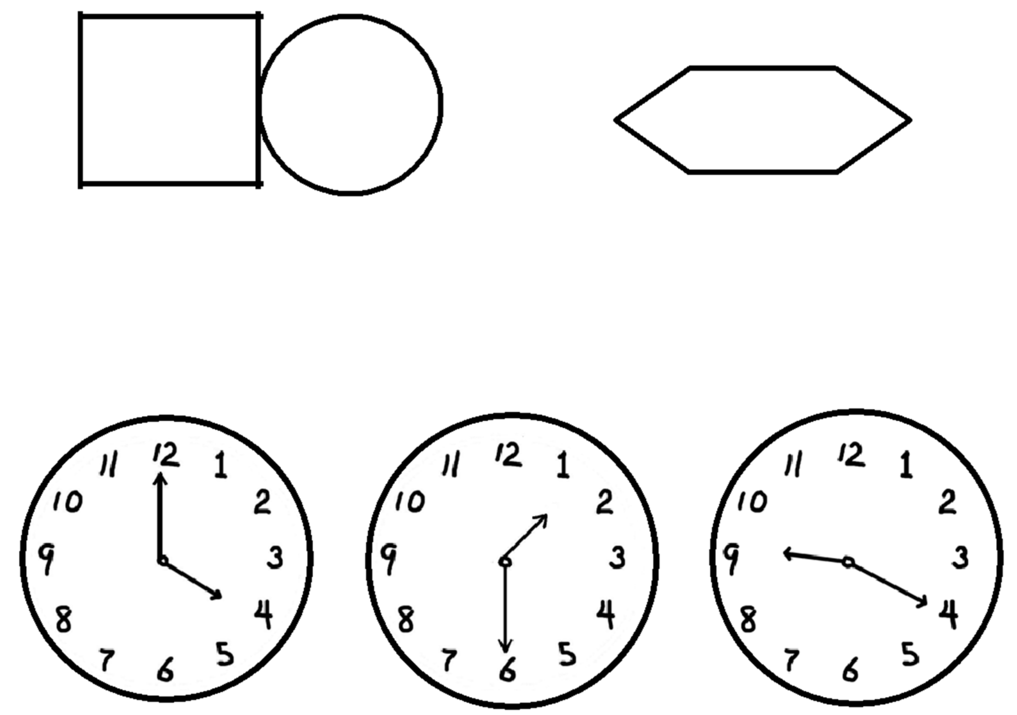

Figure S.2 The Developmental Behavior Assessment Scale for Children from 0–6 Years (Child Mental Scale-II) Copying graphs and knowing the clocks and watches

Table S.2 Basic information and result records of the Developmental Behavior Assessment Scale for Children from 0−6 Years (Child Mental Scale-II)

Name		Sex		Nation
Date of test				
Date of birth				
exact age				
Project	Mental age (months)		Development quotient (DQ)	
Gross motor				
Fine movement				
Adaptability				
Language				
Social behavior				

Tester:

Supplementary Materials B

Developmental Behavior Assessment Scale for Children from 0−6 Years (Child Mental Scale-II)

operation method and test pass requirements

(ellipsis)
